# Mechanical Versus Restrictive Kinematic Alignment in Robotic-Assisted Total Knee Arthroplasty: A Randomized Controlled Trial

**DOI:** 10.3390/diagnostics15192524

**Published:** 2025-10-06

**Authors:** Alexey V. Lychagin, Andrey A. Gritsyuk, Mikhail P. Elizarov, Andrey A. Gritsuk, Maxim Y. Gavlovsky, Konstantin K. Tomboidi, Eugene B. Kalinsky, Nahum Rosenberg

**Affiliations:** 1 Federal State Autonomous Educational Institution of Higher Education I.M. Sechenov First Moscow State Medical University of the Ministry of Health of the Russian Federation (Sechenov University), 119048 Moscow, Russia; drgaamma@gmail.com (A.A.G.); elizarovm07@gmail.com (M.P.E.); andrewgritsru@gmail.com (A.A.G.J.); gavlovsky.m@yandex.ru (M.Y.G.); tomboidi98@mail.ru (K.K.T.); kalinsky.eugene@gmail.com (E.B.K.); 2 Specialists Center, National Insurance Institute, Haifa 3309511, Israel; nahumrosenberg@sheltagen.com

**Keywords:** knee joint, robotic-assisted surgery, restrictive kinematic alignment, total knee arthroplasty

## Abstract

**Background**: Lower limb malalignment is a hallmark of knee osteoarthritis, with surgical correction techniques evolving from traditional mechanical alignment (MA) to kinematic alignment (KA) approaches. Restrictive kinematic alignment (rKA) represents a hybrid strategy combining principles from both techniques. This study evaluated short-term functional outcomes following robotic-assisted total knee arthroplasty (RoTKA), comparing MA versus rKA alignment strategies. **Methods**: This prospective, randomized, single-center study enrolled 96 patients with grade 3–4 idiopathic knee osteoarthritis (Kellgren–Lawrence classification). Patients were randomized to MA (*n* = 49, mean age 67 ± 9 years) or rKA (*n* = 47, mean age 66 ± 7 years) groups. Preoperative hip–knee–ankle (HKA) angles were 172.6° ± 1.1° and 172.9° ± 0.9° for MA and rKA groups, respectively. Outcomes were assessed using Visual Analog Scale (VAS) pain scores, range of motion (ROM), Knee Society Score (KSS), Oxford Knee Score (OKS) (primary outcome), SF-36, and Forgotten Joint Score (FJS-12). **Results**: Postoperative HKA angles were 179.5° ± 1.2° (MA) and 176.0° ± 1.5° (rKA). At 14 days postoperatively, knee ROM increased by 20.5% in the MA group and 25.7% in the rKA group, with a statistically significant 5.2% intergroup difference, indicating faster postoperative recovery (*p* = 0.008). VAS pain scores decreased by 7% in the rKA group while increasing by 13% in the MA group (*p* < 0.001). At one-year follow-up, FJS-12 scores were significantly higher in the rKA group (94.8 ± 3.2 vs. 91.9 ± 2.2, *p* < 0.001). No significant differences were observed in KSS, OKS, or SF-36 score between groups. **Conclusions**: Restrictive kinematic alignment demonstrated superior early postoperative outcomes compared to mechanical alignment in RoTKA, with significantly reduced pain and improved ROM. While one-year functional outcomes were comparable between techniques, rKA may offer advantages in the immediate postoperative period, supporting its consideration as a viable alignment strategy in robotic-assisted knee arthroplasty.

## 1. Introduction

Total knee arthroplasty (TKA) is an effective treatment for end-stage knee osteoarthritis, and procedure volumes are increasing worldwide. For example, in the USA, the number of TKA surgeries is expected to rise from 652,000 in the year 2025 to 850,000 in 2930 [1]. Outcomes depend on surgical technique, implant selection, and precise restoration of limb alignment; implant malposition that leads to postoperative malalignment worsens results and shortens prosthesis longevity [2]. Coronal alignment is critical, yet despite repeated refinements, the optimal strategy remains uncertain, and a proportion of patients remain dissatisfied after TKA [3,4]. Mechanical alignment (MA), the traditional “gold standard”, targets a neutral limb axis to distribute load evenly. Still, about 20% of patients report dissatisfaction due to factors such as stiffness, instability, occult infection, or neurogenic causes, prompting exploration of alternatives [4,5]. Kinematic alignment (KA) instead aims to preserve the pre-arthritic joint line and native axis [6]. Both MA and KA are commonly performed with “manual” instrumentation, with an accuracy of approximately ±3° [7]. The hip–knee–ankle (HKA) angle defines the limb axis; typical targets are ~180° for MA and ~177° for KA [5], and manual variability can produce postoperative HKA values outside 177–183° [7,8]. HKA directly represents the mechanical, load-bearing axis of the entire limb under physiological load, is highly reliable on long-leg imaging, avoids the biases of knee-only angles, and provides a universal reporting metric across TKA alignment strategies and technologies, hence its preferred use in TKA planning, execution auditing, and outcomes research [9].

Mechanical alignment (MA) targets a neutral hip–knee–ankle axis (~180°) using bone cuts perpendicular to mechanical axes to standardize coronal load transmission and create rectangular gaps; however, normalizing alignment can elevate and flatten the joint line, alter collateral isometry, and predispose to mid-flexion laxity despite acceptable terminal balance [10].

Restricted kinematic alignment (rKA) seeks to restore the patient’s pre-arthritic knee geometry with preoperative HKA and joint-line obliquity, while prosthetic components are within predefined safety bounds to avoid extreme varus/valgus or patellofemoral overload [6]. This limited personalization preserves joint-line height and native asymmetric laxity, thereby achieving balance with fewer releases when executed with robotic-assisted total knee arthroplasty (RoTKA) [11].

Evidence from randomized and long-term cohorts indicates kinematic strategies (including rKA) provide survivorship and patient-reported outcomes comparable to MA when applied within limits, supporting a mechanism of soft-tissue preservation without compromising fixation or wear [12].

Current practice increasingly favors personalized alignment, e.g., KA functional alignment rather than relying on the default neutral-axis method [13,14]. Despite extensive evaluations of function and survivorship, no single method has proven definitively superior [14,15]. Alignment philosophies can be grouped into systematic, patient-specific, and hybrid techniques [15]. The knee joint line usually lies 2–3° in varus relative to the mechanical axis [16]; uncertainty in estimating an individual’s pre-arthritic alignment complicates target selection [13]. Patient-specific alignment leverages anatomical variability to optimize function and longevity [13], and HKA may better reflect constitutional alignment when restoring functional axes [17,18]. Hybrid approaches such as restrictive KA (rKA) integrate MA and KA principles; in functional alignment, rKA individualizes resections, soft-tissue balance, and component positioning to bone morphology and the soft-tissue envelope [13].

Robotic-assisted TKA (RoTKA) enhances precision and personalization across MA, KA, and patient-specific plans, providing intraoperative kinematic balance feedback and data capture [19]. KA demonstrates encouraging short-term outcomes, but acceptable thresholds and long-term biomechanical effects remain debated; reports include excellent results and ~97.5% 10-year survivorship, although robust long-term evidence is still limited [14,16,20,21,22]. Ongoing research, including intraoperative imaging, continues to refine coronal alignment to optimize outcomes [23,24].

Evidence comparing MA, rKA, and TKA remains heterogeneous, with a meta-analysis showing small or inconsistent differences and, in several cases, no clear superiority on patient-reported outcome measures (PROMs) or range of motion (ROM) [25].

Comparative studies of mechanical alignment (MA) and rKA have been limited by heterogeneity in instrumentation, implants, perioperative pathways, outcomes, and follow-up windows, yielding small or inconsistent effects that are difficult to generalize [20,25]. Even in robotic series, most reports emphasize radiographic accuracy rather than isolating the clinical impact of the alignment philosophy itself, and randomized contrasts of rKA vs. MA executed on the same robotic platform are uncommon [11].

To address this gap, we conducted a single-center randomized trial that controlled for an active, autonomous CT-based robotic system, implant design, and standardized perioperative care and rehabilitation, thereby isolating the alignment strategy as the causal factor. The trial links planning-to-execution precision in coronal alignment on long-leg weight-bearing radiographs to both very early recovery and 12-month patient-reported outcomes [9].

Accordingly, considering this research gap, our objective is to compare component alignment accuracy and clinical outcomes in an autonomous robotic system using MA versus rKA protocols. We hypothesize that robotically executed rKA will yield superior early function compared with MA while maintaining implant stability.

## 2. Methods

### 2.1. Study Design

This study was designed as a single-center, prospective, parallel-group randomized trial conducted between 2023 and 2024. Ethical approval was obtained from the institutional review board prior to the initiation of the study (protocol No. 25–22; 8 December 2022), and all patients provided written informed consent in accordance with the principles outlined in the Declaration of Helsinki. The trial was registered on ClinicalTrials.gov (ID: NCT05750784). No blinding was implemented (open-label design), as the treatment surgeons and patients were aware of the alignment technique used. However, all patients received identical perioperative care and rehabilitation to ensure comparability between groups.

### 2.2. Participants

Patients scheduled for primary total knee arthroplasty (TKA) due to advanced osteoarthritis of the knee were screened for eligibility. All the patients were referred to the central medical center, where the study was conducted, from the affiliated medical centers. Inclusion criteria were adults 50–90 years of age with primary knee osteoarthritis of Kellgren–Lawrence grade 3 or 4, determined from weight bearing radiographs of knee joint, i.e., x-ray image showing moderate or large osteophytes, narrowing of joint space, sclerosis and deformity of bone margins [26], knee pain severity >3 on a 11-point Visual Analog Scale (VAS), ability to attend all follow-up visits through 12 months postoperatively, and ability to provide informed consent and comply with study procedures. Exclusion criteria: any contraindication to surgery (e.g., active infection or severe uncontrolled comorbidity), poorly controlled diabetes mellitus (glycated hemoglobin >9%), significant hematologic disorders (such as severe anemia with hemoglobin <9.0 g/dL or coagulopathy), expected inability to adhere to the postoperative rehabilitation and follow-up protocol, or patient refusal to participate.

**Figure 1:**
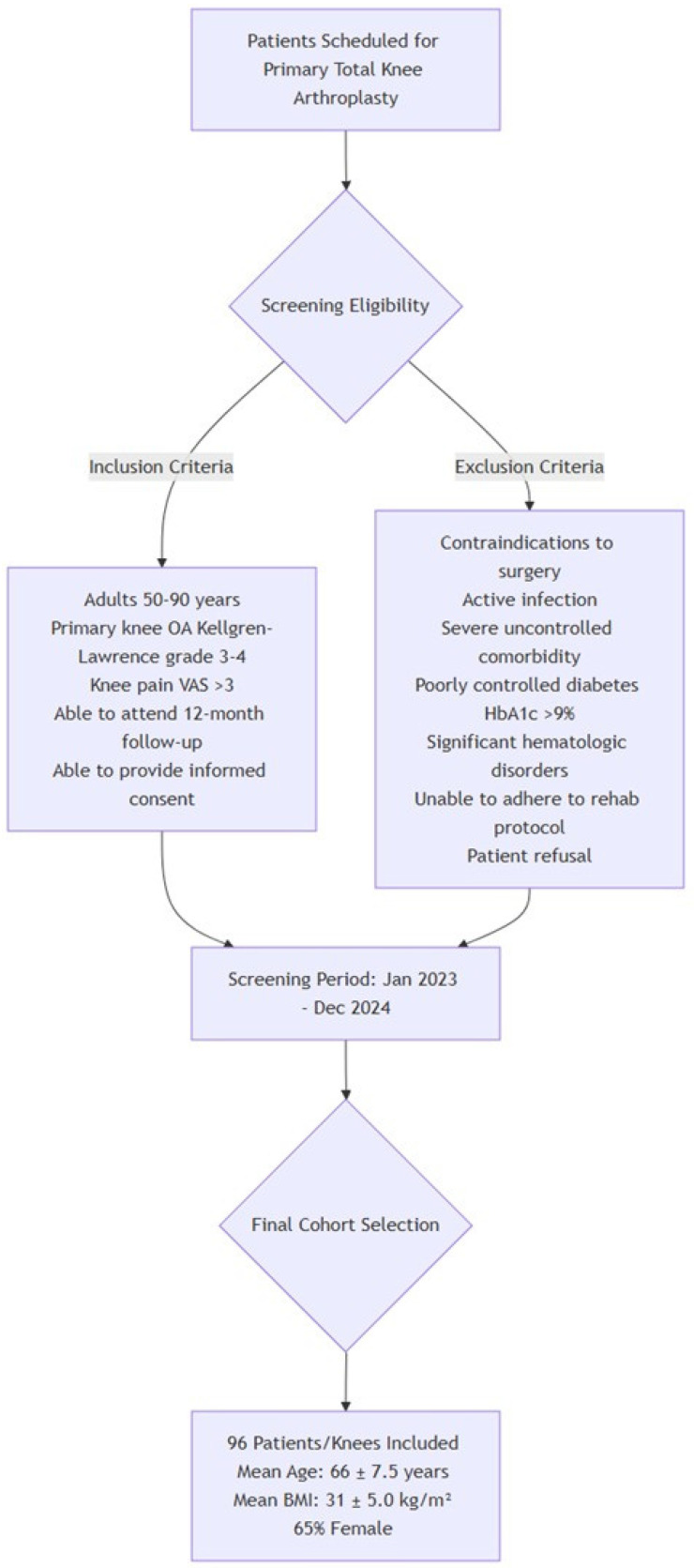
Patients’ recruitment protocol.

Consecutive patients meeting these criteria between January 2023 and December 2024 were enrolled in the study. After screening and exclusions, a final cohort of 96 patients (corresponding to 96 knees) was included in the analysis. The cohort had a mean age of 66 years (SD ± 7.5 years) and a mean body mass index (BMI) of 31 kg/m^2^ (SD ± 5.0); 65% of the patients were female (Table 1, Figure 1).

### 2.3. Randomization and Group Assignment

The patients were randomly assigned to one of two intervention groups: the mechanical alignment total knee arthroplasty (MA) Group 1 or the restrictive kinematic alignment (rKA) total knee arthroplasty Group 2. The randomization sequence was generated using a computer-based random number algorithm. Allocation assignments were concealed in sequentially numbered opaque envelopes, which were opened after the patient had given consent and was prepared for surgery. This process ensured that assignments were made unbiasedly to groups. The group allocation determined the alignment strategy used during RoTKA.

Each patient was informed of their group assignment and the nature of the alignment technique. All other aspects of perioperative management were identical between groups.

The mean age of Group 1 (49 patients) was 67± 9 (SD) years, and in Group 2 (47 patients), 66 ± 7 (SD) years. Mean BMI 30.5 ± 5.1 kg/m^2^ in Group 1 and 31.3 ± 4.7 kg/m^2^ in Group 2. The mean preoperative hip–knee–ankle (HKA) angle was 172.6° ± 1.1° in Group 1 and 172.9° ± 0.9° in Group 2 (no significant difference for these parameters, *p* > 0.05, *t*-test) (Table 1).

### 2.4. Surgical Technique

**Figure 2:**
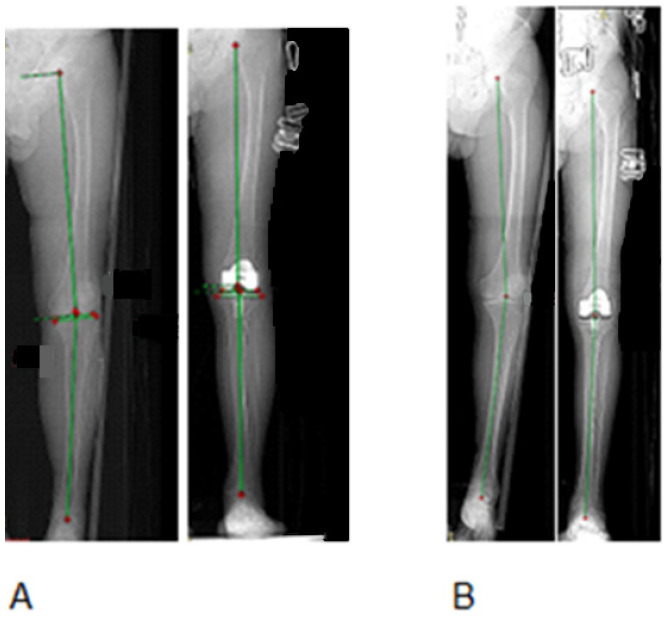
Example of MA and rKA alignments according to the HKA measurements on standing radiographs of lower limbs in two patients before and after TKA. (**A**): HKA of 173.5° was corrected to 180° (MA). (**B**): HKA of 170.7° was corrected to 178.7° (rKA). MA—mechanical alignment; rKA—restrictive kinematic alignment; TKA—Total Knee Arthroplasty; HKA—hip–knee–ankle angle.

All surgeries were performed with the assistance of an active robotic system for TKA (TSolution-One^®^ Surgical System, THINK Surgical, Inc., Fremont, CA, USA) by two experienced surgeons (A.V.L., A.A.G.). Preoperative planning and execution followed a standardized protocol. Each patient underwent imaging studies, including weight-bearing long-leg radiographs and a computed tomography (CT) scan of the entire lower limb (hip, knee, and ankle). The CT data were imported into the system’s planning software (TPLAN version 1.1, THINK Surgical, Inc., Fremont, CA, USA) to create a patient-specific three-dimensional surgical plan. This plan determines the optimal positioning, sizing, and alignment of components based on the assigned technique for each group. For Group 1 patients, the surgical plan aimed to restore the limb’s neutral mechanical axis. The femoral and tibial components were planned perpendicular to their respective mechanical axes, targeting a HKA angle of 180° (i.e., 0° deviation from neutral alignment). For the Group 2 patients, the plan was customized to approximate the patient’s native knee anatomy and limb alignment within safe limits. In practice, this meant the planned HKA angle could intentionally deviate slightly from neutral to match the pre-arthritic alignment of the limb. A mild varus or valgus alignment was accepted in the rKA group (typically up to about ±3° from neutral) to recreate the natural joint line obliquity and ligament balance. All other implant alignment parameters (such as sagittal slope and component rotation) were set according to standard principles and the patient’s anatomy, consistent across both groups (Figure 2).

All patients received the same surgical technique, aside from the alignment targets. The surgeries were executed under spinal anesthesia without the application of a tourniquet. A midline skin incision and medial parapatellar arthrotomy were used in all cases. The robotic system was utilized to perform bone resections according to the preoperative plan. After rigid fixation of tracking markers to the femur and tibia, and registration of the patient’s anatomy, the robotic cutting unit autonomously resected bone under the surgeon’s supervision to achieve the planned component positioning. The surgeon performed soft tissue releases and ligament balancing as needed. In Group 1, any necessary ligament releases were performed to achieve a neutral alignment and balanced gaps. In Group 2, soft tissue releases were minimized to preserve the patient’s natural soft tissue laxity, unless an excessive imbalance was present, as determined by the surgeon’s intraoperative impression. The same type of knee implant was used for all patients (a cemented, cruciate-substituting TKA prosthesis: Zimmer Persona PS (posterior stabilization; Zimmer, Warsaw, Indiana), to eliminate implant design as a variable. Patellar resurfacing was performed in all cases. After implantation, alignment and stability were verified, and the wound was closed in layers in the standard fashion. Postoperative management was identical for both groups. All patients received prophylactic antibiotics (Cefazolin sodium—1.0 g intravenously by drip 40 min before surgery, after surgery intramuscularly 3 times during the first 24 h) and thromboprophylaxis (Low molecular weight heparin Clexane^®^ 40 mg was administered subcutaneously 6–8 h before the operation, and rivaroxaban Xarelto^®^ 10 mg was administered the next day after TKA, and then for six weeks) per institutional protocol.

A standardized rehabilitation program was initiated on the first postoperative day, including early mobilization, weight-bearing as tolerated, and range-of-motion exercises. Patients were discharged within 5–7 days after surgery and continued supervised physiotherapy as outpatients. Compliance with rehabilitation was encouraged equally in both groups.

### 2.5. Outcome Measures

Clinical and functional outcomes were assessed using a combination of patient-reported outcome measures (PROMs), clinician-reported scores, and objective functional tests. Pain intensity was evaluated using an 11-point Visual Analog Scale (VAS), where 0 indicated no pain and 10 indicated the worst possible pain. Knee-specific function and symptoms were assessed by the Oxford Knee Score (OKS) [27], a 12-item questionnaire scored from 0 (worst) to 48 (best), and by the Knee Society Score (KSS). The KSS was recorded according to the 2011 updated version, which provides separate Knee Score and Function Score components (each scored 0 to 100 points) [28]. In addition, the Forgotten Joint Score-12 (FJS-12) was used to measure the patient’s awareness of the artificial joint in everyday life (score range 0–100, with higher scores indicating better ability to “forget” the joint) [29]. General health-related quality of life was evaluated using the 36-Item Short Form Health Survey (SF-36) [30], which yields physical and mental component summary scores normalized to a scale of 0–100. Objective physical function was tracked by measuring the knee range of motion (ROM) in degrees using a goniometer (active flexion and extension).

Baseline (preoperative) assessments for all scores and ROM were obtained within one month before surgery. Postoperative assessments were conducted 14 days after surgery (early postoperative check) and then at 3, 6, and 12 months after surgery. These follow-up intervals were chosen to represent early rehabilitation progress (2 weeks), intermediate recovery (3 and 6 months), and the definitive one-year outcome. Clinical research personnel who were not part of the surgical teams administered the PROM questionnaires and conducted ROM measurements at follow-up visits. All outcome measures were recorded at each visit, with the one-year postoperative outcomes considered the primary endpoint for between-group comparisons. Follow-up assessors were blinded to group allocation.

The primary endpoint of the study is the between-group difference in Oxford Knee Score (OKS) change from baseline to 12 months after surgery, analyzed as a continuous outcome (higher scores indicate better outcomes). The OKS is selected as the single primary endpoint because it is knee-specific, patient-centered, validated, and responsive to clinically important change after TKA; it is widely recommended for comparative effectiveness studies in knee arthroplasty and has an established minimal clinically important difference (MCID) of ~5 points [31].

### 2.6. Radiological Evaluation

Radiographic evaluation of limb alignment was performed to compare the effectiveness of the two alignment strategies. All patients underwent full-length anteroposterior radiographs of the lower extremities preoperatively and after surgery (early postoperative period and/or at the 3-month follow-up). Using these weight-bearing radiographs, the HKA angle was measured as an index of coronal alignment. The HKA angle was defined as the angle between the mechanical axis of the femur (a line from the center of the femoral head to the midpoint between the medial and lateral femoral condyles) and the mechanical axis of the tibia (a line from the center of the intercondylar notch of tibial plateau to the midpoint between the most prominent points of the ankle medial and lateral malleoli) [32]. Measurements were conducted with digital image analysis software (RadiAnt DICOM Viewer version 2022.1, Medixant, Poznań, Poland). Two experienced observers, an orthopedic surgeon and a musculoskeletal radiologist, reviewed the radiographs together in a collaborative manner to determine the HKA angle for each case. Any discrepancies in measurement were resolved by consensus during the joint review session. This collaborative review process ensured the accuracy and reliability of the radiographic measurements. The preoperative and postoperative HKA angles were recorded.

### 2.7. Statistical Analysis

The primary analysis followed the intention-to-treat principle, i.e., all randomized participants were analyzed in their allocated group, in a postoperative course [33]. The study aimed for statistical power values above 0.8, as the expected effect size was large (Cohen’s d ≥ 0.65–0.7). This range is commonly observed in evaluations of orthopedic procedure outcomes [34].

The sample size was prespecified for the primary endpoint (between-group difference in 12-month Oxford Knee Score [OKS]). We powered the trial to detect the OKS minimal clinically significant difference (MCID) of ~5 points [31] with a two-sided α of 0.05, power of 0.80, and 1:1 allocation. We assumed SD = 8–9, as 150–160% of the MCID [31]. Under these assumptions, the required size is approximately 46 participants per group (≈92 total); to allow for a small attrition margin, the target enrollment was 96 (an average of 48 participants per group), which was achieved with complete 12-month follow-up. At *n* = 48 per arm, this design provides ≥80% power to detect a 4.6–5.1-point OKS difference for SD 8–9. It yields an approximate 95% interval half-width of ±3.2 to ±3.6 points around the between-group difference, i.e., precision tighter than the OKS MCID [31,34].

A single primary endpoint (the between-group difference in 12-month OKS) was prespecified to control the confirmatory Type I error. Secondary outcomes (VAS, ROM, KSS, FJS-12, SF-36 across timepoints) were analyzed to provide supportive, exploratory evidence; accordingly, no formal multiplicity adjustment was applied to these analyses, and their *p*-values are reported as nominal and interpreted together with effect sizes, 95% confidence intervals, and clinically important difference thresholds (e.g., OKS MCID ≈5 points; proposed FJS-12 meaningful change ≈ 14–17 points) [31]. Study conclusions are based on the primary endpoint, in keeping with CONSORT recommendations for prespecified primary outcomes and exploratory secondary analyses in randomized trials (Appendix).

For comparisons between the two groups (MA vs. rKA), when the normal distribution of the groups was confirmed, the *t*-test was used. For non-normally distributed continuous outcomes, the Mann–Whitney U test was used for comparison. Categorical variables were compared using the chi-square test or, when expected cell counts were <5, Fisher’s exact test. Changes in outcome measures over time (within each group) were analyzed using a paired *t*-test for normally distributed data and the Wilcoxon signed-rank test for paired non-parametric data. A two-tailed *p*-value < 0.05 was considered to indicate statistical significance for all analyses.

## 3. Results

During the one-year follow-up, no significant postoperative complications requiring revision surgery were observed in either study group. All patients were available for follow-up, resulting in 100% one-year survivorship of the prostheses [35].

### 3.1. Pain

Preoperatively, the two groups had comparable pain levels on the visual analog scale (VAS), with an average score of 6 ± 2 (SD) in Group 1 and 7 ± 1 (SD) in Group 2 (*p* = 0.35, *t* test).

**Figure 3:**
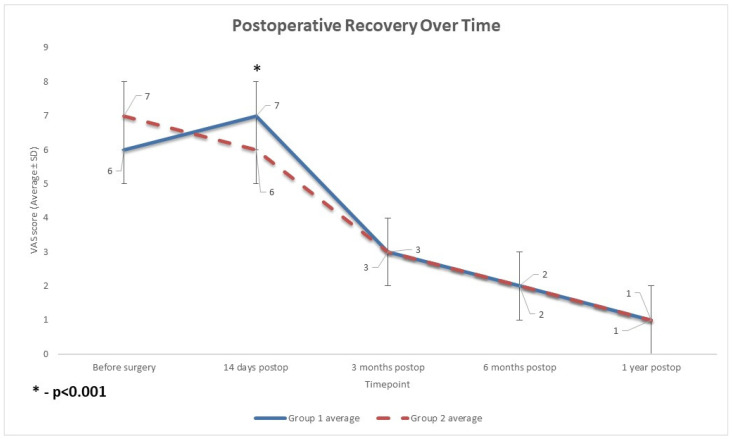
The profiles of improvement of pain levels after surgery. Both patient groups exhibited the anticipated trajectory of post-surgical pain relief, moving from severe preoperative pain to moderate pain in the early postoperative phase, and then to mild-to-minimal pain by 3 to 12 months after surgery. Group 1 (MA TKA) experienced a brief period of higher pain shortly after surgery compared to Group 2 (rKA TKA) (*p* < 0.001), but by 3 months, both groups had converged to equivalent pain levels. MA: mechanical alignment; rKA: restrictive kinematic alignment; TKA: Total Knee Arthroplasty; postop: postoperative.

On postoperative day 14, however, Group 2 patients experienced significantly less pain than Group 1. Mean VAS score in Group 1 increased to 7 ± 1 (SD) (13% increase). In contrast, in Group 2, the mean score decreased to 6 ± 1(SD) (a 7% decrease), yielding an intergroup difference of 14% (*p* < 0.001, *t* test) (Figure 3). Thus, in the early postoperative period, the rKA cohort reported a lower level of pain. However, by three, six, and 12 months after surgery, the VAS scores in both groups decreased to the same level as the average score of 3, 2, and 1, respectively, with no statistically significant differences between Group 1 and Group 2 (*p* = 0.4, *t* test, Figure 3).

### 3.2. Radiographic Alignment

**Figure 4:**
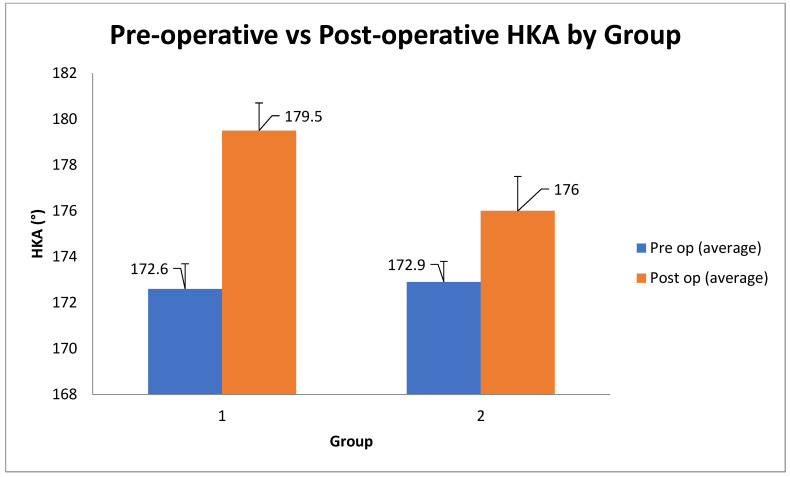
Alignment Outcomes (according to the hip–knee–ankle (HKA) alignment between Group 1 and Group 2. Group 1(MA TKA) achieved a closer alignment to the neutral 180° position (179.5° ± 1.2°) than Group 2 (rKA TKA; 176.0° ± 1.5°). The degree of correction attained in Group 1 (~7°) was more than double that of Group 2 (~3°), as planned by the study design. Pre op: before surgery Post op: after surgery. MA: mechanical alignment; rKA: restrictive kinematic alignment; TKA: Total Knee Arthroplasty; post op: postoperative; HKA: hip–knee–ankle angle.

Postoperative limb alignment differed markedly between the two techniques (Figure 4). Group 1 achieved a nearly neutral hip–knee–ankle (HKA) axis (mean 179.5° ± 1.2° SDE), whereas in Group 2, the rKA approach resulted in a slight residual varus alignment (mean 176.0° ± 1.5°). This ~3.5° difference in final HKA angle was statistically significant (*p* < 0.001, *t*-test) and reflects the intended alignment philosophies of each method. Preoperatively, both groups had similar varus deformities (mean HKA ~173°, *p* > 0.05, *t*-test). Thus, mechanical alignment produced greater coronal correction toward neutral, while rKA intentionally left a mild constitutional varus.

### 3.3. Range of Motion

**Figure 5:**
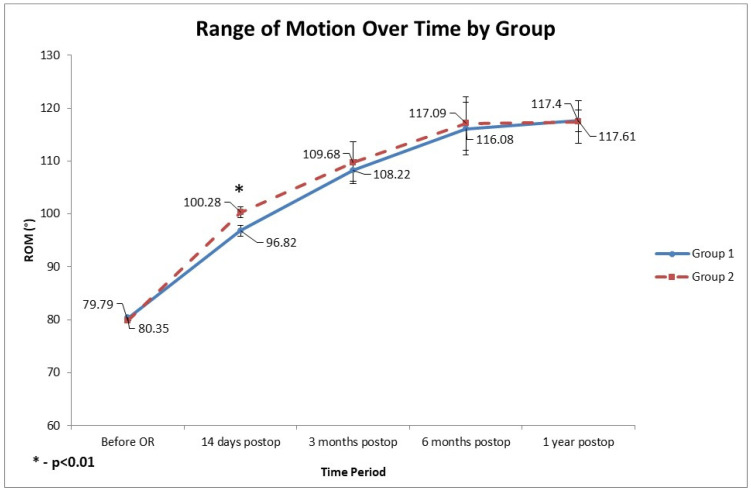
Recovery of knee range of motion (ROM) over time in two patient groups. Both groups exhibit typical post-TKA recovery patterns, characterized by a rapid gain in the first 3 months, slower progress until 6 months, and then a plateau. ROM increased by ~37° in both groups over 12 months. The early postoperative ROM advantage in the rKA TKA Group 2 (14 days after surgery, *p* < 0.01) did not persist, and both groups achieved nearly identical final ROM at one-year follow-up (~117°). OR: surgery; MA: mechanical alignment; rKA: restrictive kinematic alignment; TKA:Total Knee Arthroplasty; postop: postoperative.

Preoperatively, mean active flexion was restricted to approximately 80° in both Group 1 and Group 2 (normal active knee flexion is ~ 135° [36]). Knee range of motion improved substantially after surgery in both groups. Early postoperative flexion (14 days postoperatively) was slightly but significantly greater in the rKA cohort (average 100.28° ± 4.72° SD in Group 2 vs. 96.82° ± 7.41° SD in Group 1, *p* = 0.008) (Figure 5). However, this early advantage for Group 2 was not sustained at later follow-ups. At 3 months, 6 months, and 1 year, there were no significant intergroup differences in ROM; both groups achieved a mean flexion of approximately 120° by 1 year.

### 3.4. Functional Scores

**Figure 6:**
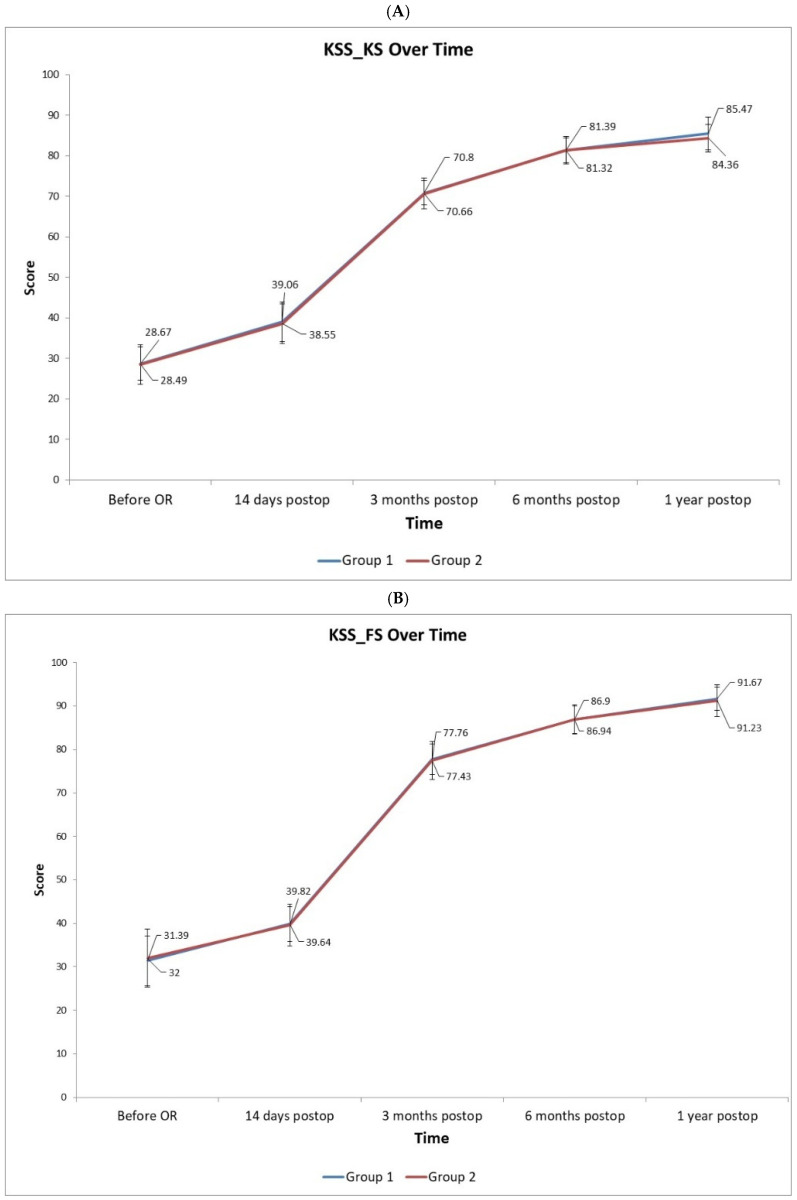

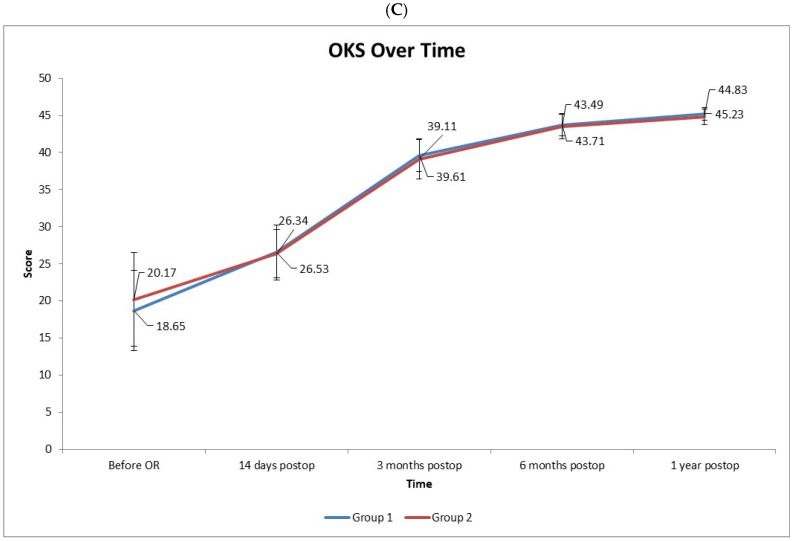
The profiles of KSS (Knee Score) (**A**), KSS_FS (Functional Score) (**B**), and OKS (Oxford Knee Score) (**C**) demonstrate considerable, clinically significant improvements from before surgery to 1 year after surgery in both patient groups. The scores rapidly increase by the 3-month and continue to gain slightly through 6 months to 1 year. There was no difference in score profiles between the two study groups (*p* > 0.05). OR: surgery. Postop: after surgery.

Postoperative functional outcomes improved in both groups with no between-group differences (Figure 6). The Knee Society Scores, both the Knee Score (KSS-KS) and Function Score (KSS-FS), increased substantially from baseline in Groups 1 and 2, and did not differ significantly between the two cohorts at 3 or 6 months. The Oxford Knee Score (OKS) likewise showed parallel improvement in both groups through 1 year, with no statistically significant group differences at early or mid-term follow-ups. By 12 months postoperatively, OKS in Group 2 was marginally higher than in Group 1 (mean difference ~0.4 points; Group 2 44.3 vs. Group 1 43.9, *p* = 0.046, *t*-test), reaching marginal statistical significance. Still, this difference is very small in absolute terms, indicating that clinically, the two alignment strategies provided equivalent knee function at 1 year.

### 3.5. Quality of Life and Satisfaction Scores

**Figure 7:**
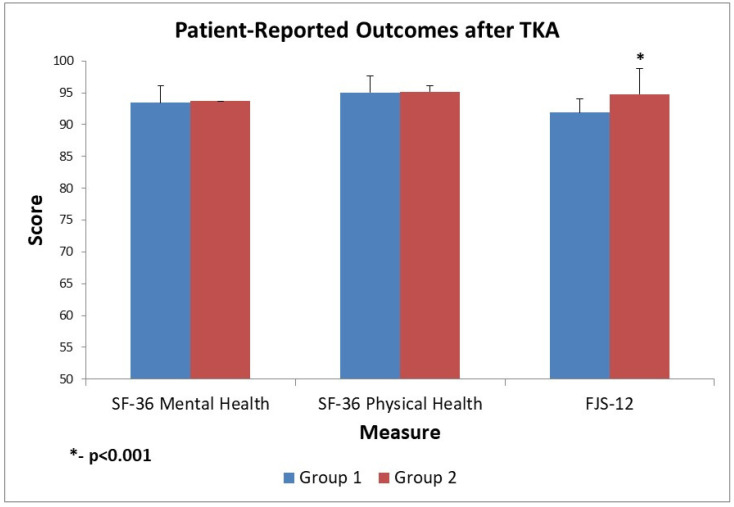
Patient-Reported Outcome Scores after Total Knee Arthroplasty (TKA), Group 1 (MA TKA) vs. Group 2 (rKA TKA). Both groups exhibit excellent outcomes post-TKA: SF-36 Physical Health scores are ~95 in both groups, indicating nearly optimal physical function, and FJS-12 scores are above 90, suggesting that patients generally do not notice their replaced knees in daily life. The differences between Group 1 and Group 2 are minimal (e.g., ~0.2 points in SF-36 Mental, ~0.1 in SF-36 Physical, and a slightly larger significant 2.9 points in FJS-12, favoring Group 2—*p* < 0.001). MA—mechanical alignment; rKA—restrictive kinematic alignment; TKA—Total Knee Arthroplasty; postop—postoperative.

Patient-reported quality of life and satisfaction measures were similar between groups (Figure 7). The SF-36 scores (both Physical and Mental Component Summary) improved postoperatively in both Group 1 and Group 2, with no significant intergroup differences at any assessed timepoint. At one year, the mean SF-36 Physical Component was nearly identical between groups (no statistically significant difference, *p* > 0.05, *t*-test), indicating comparable general health status and recovery. The Forgotten Joint Score-12 (FJS-12) did reveal a notable difference at the final follow-up. By 12 months, Group 2 reported a significantly higher FJS-12 (94.8 ± 3.2) than Group 1 (91.9 ± 2.1; *p* < 0.001), suggesting that patients were more likely to “forget” the artificial joint in everyday life.

Aside from the FJS, other patient-reported outcome measures showed no clear advantage to either alignment technique. Both cohorts achieved high postoperative satisfaction and returned to function by one year.

## 4. Discussion

This study compared the outcomes of MA and rKA in robotic-assisted total knee arthroplasty (RoTKA). The robotic platform enabled precise execution of preoperative planning, resulting in high alignment accuracy in both groups. The final postoperative HKA angle was within ±1.2° of the planned 180° in the MA group and ±1.5° of the planned 177° in rKA group, consistent with previous findings demonstrating the high reproducibility and precision of robotic-assisted alignment strategies in total knee arthroplasty [37,38].

Our results indicate that both alignment techniques yielded substantial functional improvements over the baseline. While long-term clinical outcomes were statistically equivalent between groups, patients in the rKA group demonstrated superior early postoperative outcomes. Specifically, on day 14 post-surgery, rKA patients reported significantly lower pain scores and greater ROM compared to the MA group. These findings are congruent with prior investigations suggesting that kinematic alignment offers improved early recovery and reduced discomfort [39,40]. The magnitude of the initial ROM difference was small, and its disappearance over time suggests minimal clinical impact. This is consistent with previous evidence indicating that kinematic alignment in TKA yields only a slight increase in postoperative knee flexion, typically on the order of a few degrees, when compared to MA [41].

The functional outcomes for Group 1 and Group 2 were nearly identical across all follow-up intervals, suggesting that the surgical technique differences (e.g., mechanical vs. restricted kinematic alignment) did not result in a measurable difference in recovery as assessed by the Knee Society Score (KSS) and Oxford Knee Score (OKS). These findings are consistent with prior studies, which report that most patients undergoing TKA experience substantial functional improvements, as indicated by KSS and OKS within the first two postoperative years [41,42].

Waterson et al. [43] reported similar short-term benefits of kinematic alignment performed using patient-specific instrumentation. The high accuracy of the robotic system used here is supported by Vaidya et al. [44], who found deviations greater than ±3° in only 3.1% of cases.

Miura et al. [45] concluded in their systematic review that kinematic yields greater postoperative ROM than mechanical alignment, but with minimal clinical relevance. This was observed in the data presented, where a 3.5° advantage in ROM was noted on day 14 in the rKA group, although this difference diminished by 3 months.

A meta-analysis by Liu et al. [46] further supports the notion that kinematic alignment may offer superior functional outcomes, particularly in terms of knee flexion. However, no overall difference was found between the two surgical methods. Our findings are directionally consistent, though we observed only a marginal and statistically insignificant difference in functional scales by one year.

Kafelov et al. [47] also compared robotic functional positioning to traditional kinematic alignment, reporting higher FJS-12 scores in the robotic group but no differences in KSS, consistent with our observation of significantly higher FJS-12 values in the rKA group (94.8 vs. 91.9, *p* < 0.001) despite equivalent KSS and OKS outcomes. This study’s results show an average 2.9-point higher FJS-12 in rKA than MA at the 12-month follow-up, which is statistically significant but below the accepted clinical thresholds: MCID ≈ 13.7–16.6 points [48]. Therefore, a 2.9-point average difference is unlikely to be clinically meaningful. This fact supports the overall conclusion that there is no difference in 12-month outcome between ME and rKA methods for TKA.

Therefore, the present data, along with other published reports, suggest that rKA may facilitate a more favorable early postoperative course without compromising one-year functional outcomes. The adoption of robotic assistance has enhanced alignment accuracy across both alignment philosophies, enabling precise replication of patient-specific surgical plans and possibly reducing complications associated with outliers in component positioning. This is of special importance because over the past decade (since 2010), robotic assistance in TKA has matured from early integration into routine workflow with high positioning accuracy to broader clinical evaluation in randomized trials and meta-analyses [37,38,44].

The fact that rKA delivers earlier ROM recovery is due to native-like joint line restoration and fewer tissue releases; however, the final ROM is governed by implant geometry, soft-tissue remodeling, and standardized rehabilitation, explaining why the benefit is often transient and not durable at 12 months [25].

Despite these insights, this study is subject to several limitations. The study was conducted at a single center with a moderate sample size and a relatively short follow-up duration. Moreover, only one robotic platform was evaluated, and no subgroup analysis was performed based on implant type or patient-specific anatomical factors. Longer-term data are needed to assess prosthetic survivorship, complication rates, and revision outcomes between alignment strategies. Additionally, intraoperative decisions to perform tissue releases and their extent in the rKA arm were based on the operating surgeon’s clinical judgment of excessive imbalance, rather than on a predefined quantitative threshold. This introduces subjectivity and may permit performance and detection bias. Although this reflects current practice, there is a lack of universally accepted numeric criteria for soft-tissue balancing in TKA [15]. Future studies should prespecify objective thresholds (e.g., gap measurements, load-sensing targets) to further reduce bias. Finally, we did not capture the number of patients screened who failed to meet the inclusion criteria. The absence of this screening data limits transparency regarding the eligible population and may hinder appraisal of selection processes and external validity; future trials should prospectively document the full screening log in accordance with CONSORT guidance.

Nevertheless, the consistency of our findings with other reports reinforces the conclusion that robotic systems provide a robust tool for executing both mechanical and kinematic alignment strategies. As implant technology and robotics evolve, the integration of new alignment standards with precision-guided tools may enable greater personalization in arthroplasty planning, ultimately improving patient satisfaction and function.

According to the presented results, rKA appears suitable for patients with correctable, moderate deformity and competent ligaments who prioritize faster early recovery. Conversely, MA may be preferable in severe or complex deformity, ligament insufficiency, or when neutral alignment and reproducibility are essential. Cost-effectiveness depends on whether rKA’s early gains consistently translate into measurable reductions in resource use (e.g., length of stay, rehabilitation, analgesia). This issue should be further investigated in future studies with long-term follow-up with larger patient cohorts.

## 5. Conclusions

Restrictive kinematic alignment (rKA) in robotic-assisted total knee arthroplasty demonstrated statistically significant early advantages over mechanical alignment, including a 7.1% reduction in pain and a 4° greater range of motion at 14 days postoperatively. These early benefits did not translate into differences in the further early outcomes, with comparable functional scores, patient satisfaction, and radiographic alignment achieved in both groups by 12 months. These findings support the safety and efficacy of both alignment strategies when implemented via robotic systems, suggesting that the choice of alignment can be tailored to individual patient anatomy and expectations.

The strength of this study lies in its multifactorial assessment, encompassing pain, range of motion, satisfaction, and radiographic alignment, which establishes strong validity. This approach enhances generalizability and enables individualized alignment selection without compromising safety, while simultaneously highlighting the early recovery advantages of rKA.

This study’s results reduce the prior heterogeneity of reported outcomes following RoTKA by using a randomized, intention-to-treat design that isolates the effect of prosthesis alignment. Tracking recovery to 12 months showed that rKA’s early advantages diminish by one year. These results turn suggestive evidence into a precise, unbiased estimate of rKA versus MA in the early postoperative course, directly informing clinical decisions and patient counseling, which should be further supported by the long-term follow-up studies.

## Figures and Tables

**Table 1:** Characteristics of the study groups.

	Group	*n*	Average	Standard Deviation	*p* (*t*-Test)
Age (years)	1	49	67	9	0.58
	2	47	66	7	
Female	1	36 (73%)	-	-	
	2	35 (74%)	-	-	
Male	1	13 (27%)	-	-	
	2	12 (26%)	-	-	
BMI (kg/m^2^)	1	49	30.45	5.07	0.39
	2	47	31.31	4.65	
HKA prior operation (°)	1	49	172.61	1.08	0.14
	2	47	172.91	0.88	

## Data Availability

The original contributions presented in this study are included in the article/Supplementary Materials. Further inquiries can be directed to the corresponding author.

## Supplementary Materials

The following supporting information can be downloaded at: https://www.mdpi.com/article/10.3390/diagnostics15192524/s1.
